# Interventions in women with type 2 diabetes mellitus in the pre‐pregnancy, pregnancy and postpartum periods to optimise care and health outcomes: A systematic review

**DOI:** 10.1111/dme.15474

**Published:** 2024-11-11

**Authors:** Sowmiya Gunabalasingam, Artemis Kyrka, Lily Hopkins, Rivka Lebrett, Eleanor Dyer, Rita Forde, Nicola Heslehurst, Claire L. Meek, Danielle A. J. M. Schoenaker, Angela C. Flynn, Sara L. White

**Affiliations:** ^1^ Department of Women and Children's Health School of Life Course and Population Sciences, King's College London London UK; ^2^ Department of Non‐Communicable Disease Epidemiology, Faculty of Epidemiology and Population Health London School of Hygiene and Tropical Medicine London UK; ^3^ Clalit Health Services Rehovot Israel; ^4^ Salford Royal Hospital, North West of UK Foundation School Salford UK; ^5^ Population Health Sciences Institute Newcastle University Newcastle upon Tyne UK; ^6^ Faculty of Nursing, Midwifery and Palliative Care King's College London London UK; ^7^ School of Nursing and Midwifery University College Cork Cork Ireland; ^8^ Leicester Diabetes Centre and Leicester NIHR Biomedical Research Centre University of Leicester, Leicester General Hospital Leicester UK; ^9^ School of Human Development and Health, Faculty of Medicine University of Southampton Southampton UK; ^10^ MRC Lifecourse Epidemiology Centre University of Southampton Southampton UK; ^11^ NIHR Southampton Biomedical Research Centre University of Southampton and University Hospital Southampton NHS Foundation Trust Southampton UK; ^12^ School of Population Health, Royal College of Surgeons in Ireland Dublin Ireland; ^13^ Department of Nutritional Sciences School of Life Course and Population Sciences, King's College London London UK; ^14^ Department of Diabetes and Endocrinology Guy's and St Thomas' Hospital NHS Foundation Trust London UK

**Keywords:** intervention, postpartum, pregnancy, pre‐pregnancy, type 2 diabetes

## Abstract

**Aims:**

Type 2 diabetes is a chronic condition affecting increasing numbers of women of reproductive age. Recent UK data show more severe adverse offspring outcomes (stillbirth, neonatal death) than in infants of those with Type 1 diabetes. This systematic review aimed to evaluate randomised controlled trials (RCTs) undertaken in the pre‐pregnancy, pregnancy and the postpartum periods in women with Type 2 diabetes, to optimise care and health outcomes.

**Methods:**

Six electronic databases were searched for eligible studies from January 2000 to September 2023; eligibility included RCTs of behavioural components, supplementation, pharmacotherapy and/or medical devices. Studies were screened in duplicate, and data were extracted on outcomes including behavioural, anthropometry, clinical measures and maternal and offspring outcomes. A narrative synthesis was performed.

**Results:**

Eleven trials (12 papers) were included (total 1356 women with Type 2 diabetes, *n* = 25–502). Ten interventions were conducted in pregnancy, and one in the postpartum period. No pre‐pregnancy RCTs were identified. Interventions included pharmacotherapies and supplementation, a diabetes‐specific antenatal programme, continuous glucose monitoring and postpartum exercise. We found a paucity of interventions limited by inadequate design, statistical power and poor reporting. The largest Type 2 diabetes pregnancy study to date demonstrated evidence of benefit for adding metformin to a standard insulin regimen compared to insulin alone. Other interventions need replication in larger studies among more diverse groups.

**Conclusion:**

This review identified few RCTs targeting women of reproductive age with Type 2 diabetes particularly lacking in the preconception and postpartum periods. Tailored pre‐pregnancy, pregnancy and postpartum interventions for women with Type 2 diabetes to optimise care and health outcomes are urgently needed.

**What's new?**

- Serious adverse pregnancy outcomes including stillbirth and neonatal death are higher among infants of women with Type 2 diabetes compared with infants of those with Type 1 diabetes and the background population.
- This systematic review identifies a lack of well‐designed, adequately powered RCTs designed to improve outcomes for women with Type 2 diabetes during the pre‐pregnancy, pregnancy and postpartum periods.
- High‐quality trials specifically designed for women with Type 2 diabetes are urgently needed to identify interventions in the pre‐pregnancy, pregnancy and postpartum periods that improve outcomes for this group.

## 1 INTRODUCTION

In line with the global prevalence of diabetes, the number of pregnancies complicated by Type 2 diabetes is increasing. In England and Wales in 2021/2022, 55% of women with diabetes in pregnancy were reported to have Type 2 diabetes, many of whom face health inequalities before and during pregnancy.^1^ Infants of women with Type 2 diabetes in pregnancy are at greater risk of adverse outcomes including perinatal and neonatal death, compared to those of women with Type 1 diabetes.^2^ Diabetes in pregnancy can also increase the risk of small and large for gestational age (SGA and LGA respectively), gestational hypertension, pre‐eclampsia and pre‐term delivery.^3^

To optimise pregnancy preparation and outcomes, current guidelines advise intervention in the pre‐pregnancy period for women with Type 2 diabetes in the United Kingdom (UK), including achieving an HbA1c ≤48 mmol/mol (6.5%), high dose folic acid (5 mg) supplementation, identification and management of diabetes complications and hypertension, and ceasing potentially teratogenic medication.^4^ However, multiple factors impact this population's ability to effectively prepare for pregnancy^5^ and implementation of this advice has thus far proven suboptimal with only around 10% of women with Type 2 diabetes meeting these targets.^1^ Furthermore, we previously reported a high proportion of obesity (65%), smoking (23%) and illicit/recreational drug use (9%) among women with Type 2 diabetes planning pregnancy compared to women without diabetes.^6^ Together, this evidence highlights the significant challenges to pre‐pregnancy care, demonstrating the need for targeted interventions in the pre‐pregnancy period for women with Type 2 diabetes.

In pregnancy, women with Type 2 diabetes are typically advised to conduct self‐monitoring of blood glucose levels, with additional advice on diet and exercise, and treatment with metformin and/or insulin to optimise blood glucose management.^4^ Insulin requirements vary through pregnancy and during delivery. Between 16 and 37 weeks' gestation, an increase in insulin resistance means that the daily insulin requirement can almost double that of the pre‐pregnancy requirement.^7^ A largely uniform approach to the use of metformin and insulin in the management of Type 2 diabetes in pregnancy has resulted in a lack of studies evaluating optimal dosing or regimens that may optimise glycaemia. Furthermore, most studies evaluating the efficacy of behaviour change interventions in pregnancy are tailored towards gestational diabetes,^8^, ^9^, ^10^ highlighting the need to conduct interventions that may improve pregnancy outcomes in women with Type 2 diabetes.

UK guidelines on postpartum care for Type 2 diabetes focus on medication management immediately after birth to achieve/optimise glycaemia, advising on nutrition and advocating breastfeeding. UK guidelines recommend that women should also be referred back to their routine care providers in primary care, where advice should be given on planning for future pregnancies including appropriate contraception.^4^ However, there are a paucity of interventions during this period, and limited data on the effectiveness of guidance to inform the optimal management of women with Type 2 diabetes during the postpartum period and beyond.

Due to the increasing rates and complications associated with Type 2 diabetes among women of reproductive age, it is particularly important for women to receive evidence‐based optimal management before, during and after pregnancy. There has been no detailed review of interventions essential for translation and implementation into clinical practice; this is necessary as a benchmark of the current best approach, and to highlight potential inadequacies in the evidence and to drive innovative practice. As part of a series of systematic reviews formulated to identify the evidence for optimising care and health outcomes, this systematic review aimed to evaluate randomised controlled trial (RCTs) interventions in the pre‐pregnancy, pregnancy and postpartum periods for women with Type 2 diabetes.

## 2 METHODS

The protocol for this series of systematic reviews was registered in the PROSPERO database (CRD42021292405). Collectively, these reviews aimed to assess the optimal management of women with Type 2 diabetes in pre‐pregnancy, pregnancy and postpartum periods, encompassing interventional, observational and qualitative study designs. The present review focuses on RCT interventional studies in women with Type 2 diabetes before, during and after pregnancy, and has been reported in accordance with the PRISMA guidelines.^11^

### 2.1 Inclusion and exclusion criteria

Inclusion and exclusion criteria for this review were developed using a PICOS (population, intervention, comparison, outcomes, study design) framework described in Table 1. Studies were included if they met the following criteria: (1) women of reproductive age between 18 and 50 years living with Type 2 diabetes in the pre‐pregnancy, pregnancy and postpartum periods; (2) interventions based on behavioural components (e.g. diet and physical activity), supplementation (e.g. folic acid), pharmacological treatment (e.g. oral diabetes medication and insulin) and technology (e.g. glucose sensors, smartphone applications, remote monitoring and insulin pumps); (3) comparison group including no intervention, standard care, placebo and dose comparator; (4) data reporting outcomes for mother and newborn (e.g. behavioural, clinical and pregnancy outcomes); (5) RCT studies that targeted women with Type 2 diabetes or ‘overt diabetes’ diagnosed in early pregnancy through identification of marked hyperglycaemia were included. Studies meeting the following criteria were excluded: (1) qualitative or observational study designs; (2) abstracts and reviews; (3) women aged less than 18 years or more than 50 years; (4) studies not published in the English language; (5) studies that included women with Type 2 diabetes along with other populations but did not report disaggregated outcome data for the Type 2 diabetes population.

**TABLE 1:** PICOS framework used for the systematic review.

Selection criteria	Inclusion criteria
P‐Population	Women of reproductive age living with Type 2 diabetes, which was diagnosed prior to pregnancy
I‐Intervention	Behavioural (e.g. diet and physical activity), technological (e.g. sensors, apps, remote monitoring, accelerometer and insulin pumps), pharmacotherapy (e.g. oral agents and insulin types) and supplementation (e.g. folic acid)
C‐Comparison	Placebo, no intervention, dose comparator
O‐Outcome	Behavioural (e.g. diet, physical activity and adherence to treatment), anthropometry and clinical measures (e.g. blood pressure), clinical outcomes (e.g. maternal and offspring, e.g. macrosomia, mode of delivery, hypoglycaemia) and biochemical measures (e.g. HbA1c)
S‐Study design	Randomised controlled trials

### 2.2 Literature search

The electronic databases MEDLINE, EMBASE, CINAHL, PsycINFO, ASSIA and the Cochrane library were searched first in February 2022 with a repeat updated search in September 2023. Search strategies are shown in Data S1. The search including human studies was originally unlimited by date, but a cut‐off of the year 2000 was added as an exclusion criteria at the screening stage through group discussion and agreement, in view of the much‐changed landscape of Type 2 diabetes pregnancy prevalence and management options. A systematic database search was conducted using a combination of Medical Subject Headings (MeSH) terms, listed in Data S1, using a Boolean combination, different for each electronic database. Some MeSH terms commonly used included #prepregnancy, #pregnancy, #perinatal, #prenatal, #postpartum, #Type 2 diabetes mellitus, #Type 2 diabetes and #diabetes mellitus adult onset. Backward and forward citation searching was undertaken using a citation chasing tool (R Shiny)^12^ following identification of eligible studies. As this systematic review was part of a wider series, searching and selection were conducted for all reviews concurrently.

### 2.3 Study selection

The results of the literature search were exported to EndNote version 20 to remove duplicates with the remaining articles imported into Covidence systematic review software for title and abstract screening. Titles and abstracts were independently screened against the eligibility criteria, in duplicate, by all authors. If eligibility was unclear through title and abstract alone, full text articles were screened. Any disagreements between reviewers were resolved through consensus opinion.

### 2.4 Data extraction

A standardised template for data extraction was created which included title, authors, publication date, study aim and design, country of study, intervention details including period of intervention (i.e. pre‐pregnancy, pregnancy or postpartum), sample size, participant characteristics and study outcomes. Data extraction was performed by one author (SG) and validated by a second author (ACF). Any disagreements were resolved by reaching a consensus opinion with a third author (SLW).

### 2.5 Main outcomes

Data were extracted on outcomes available pertaining to women with Type 2 diabetes. These included behavioural aspects such as diet, physical activity and adherence to management; anthropometry (e.g. BMI and body composition); clinical measures (e.g. blood pressure (BP); HbA1c); and maternal and offspring clinical outcomes (e.g. macrosomia, mode of delivery and perinatal weight).

### 2.6 Data synthesis

Due to heterogeneity of study design, interventions and outcomes, conducting a meta‐analysis of effect estimates was not appropriate. Therefore, a narrative synthesis, divided into studies conducted in the pre‐pregnancy, pregnancy and postpartum periods, was conducted in line with the synthesis without meta‐analysis (SWiM) guidelines.^13^

### 2.7 Quality assessment

The quality of the included studies was assessed using the Cochrane Risk of Bias tool for randomised trials (RoB 2).^14^ The RoB 2 domains evaluate the reliability of the randomisation process, the intervention itself and the analysis and reporting of outcomes. These domains specifically assess risk of bias arising from; the randomisation process including concealment of the allocation sequence and differences in baseline characteristics; deviation from the intended interventions such as blinding of participants and non‐adherence (selection bias); missing outcome data (attrition bias); measurement of the outcome including blinding of the outcome assessors; and selection of the reported result such as not using a pre‐specified analysis plan. Depending on answers to checklist questions informed by the RoB 2 algorithm for each domain, studies are categorised into low, medium or high quality. Quality assessment was performed by one author (SG) and validated by another author (AF). The overall risk of each study was deemed as either ‘low risk of bias’, ‘some concerns’ or ‘high risk of bias’.

## 3 RESULTS

**FIGURE 1:**
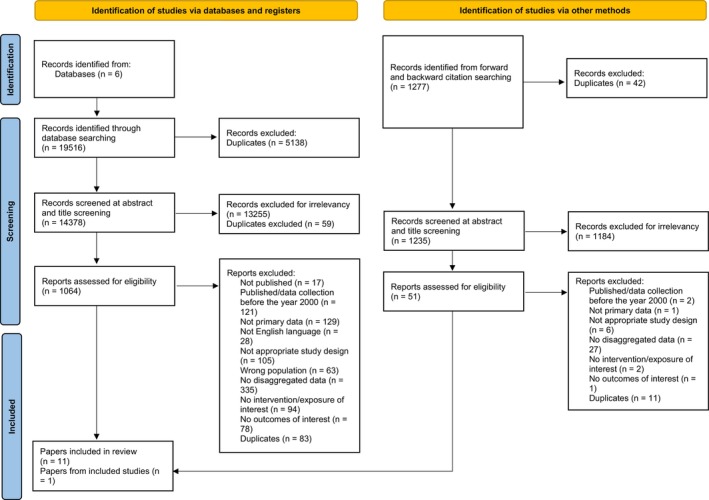
PRISMA flow diagram.

A total of 19,516 studies were identified in the electronic database search (Figure 1). After the removal of duplicates (*n* = 5138), 14,378 titles and abstracts were screened, with 1064 full texts identified as eligible for inclusion for full text screening. After removal of those eligible for inclusion in the observational and qualitative systematic reviews, 10 RCTs (11 papers) met criteria for inclusion, with one further RCT identified from reference and citation searching, totalling 11 RCTs (equating to 12 studies) included in the final synthesis.^15^, ^16^, ^17^, ^18^, ^19^, ^20^, ^21^, ^22^, ^23^, ^24^, ^25^, ^26^

### 3.1 Characteristics of included RCTs

Characteristics of the included studies, published between 2012 and 2023, are summarised in Data S2. Three RCTs were conducted in the United States,^15^, ^19^, ^23^ one RCT (two publications) in Australia/Canada,^25^, ^26^ one study in each of the United Kingdom,^16^ Denmark,^17^ Pakistan,^18^ Netherlands and Belgium,^20^ Thailand,^22^ China^24^ with the final study conducted in an unknown location.^21^ Eleven studies were conducted during the pregnancy period and assessed the following interventions: metformin and/or insulin therapy,^15^, ^18^, ^19^, ^25^, ^26^ docosahexaenoic acid‐enriched (DHA) fish oil supplementation,^16^ continuous glucose monitoring (CGM),^17^, ^20^, ^24^ moderate aerobic exercise^21^ and a diabetes group prenatal care programme.^23^ One postpartum RCT was identified that examined the use of tai chi qigong.^22^ No RCTs in the preconception period were identified. Sample sizes across the intervention studies ranged from 25^19^ to 502 participants,^25^ with 8 out of 11 RCTs including fewer than 200 participants equating to a total of 1738 women of whom 1356 had Type 2 diabetes. The recruitment gestational age during pregnancy ranged from 5.1^16^ to 27.1 weeks,^23^ with ages of participants ranging from 18 to 45 years old. Seven studies focused solely on women with Type 2 diabetes,^16^, ^19^, ^21^, ^22^, ^24^, ^25^, ^26^ with the others including mixed populations including gestational diabetes, Type 1 diabetes and individuals without diabetes.

### 3.2 Interventions

#### 3.2.1 Pregnancy interventions

Four studies investigated the effect of metformin or insulin therapies on glycaemic control and maternal and neonatal outcomes.^15^, ^18^, ^19^, ^25^ Feig et al and Refuerzo et al included women with Type 2 diabetes only whilst Fishel et al and Ainuddin et al also included women with overt diabetes in pregnancy (diagnosed before 20 weeks' gestation) (Data S3).

Fishel et al (*n* = 108) randomised women with Type 2 diabetes before 21 weeks' gestation to receive insulin detemir or NPH insulin (neutral protamine Hagedorn) in a comparative effectiveness trial.^15^ The composite primary outcome (one or more of shoulder dystocia, LGA, neonatal intensive care unit (NICU) admission, neonatal hypoglycaemia or respiratory distress) was reduced in those taking detemir compared to NPH (87% posterior probability reduction; Bayesian posterior adjusted relative risk reduction 0.88, 95% credible interval 0.61–1.12).^15^ Maternal hypoglycaemic events and hypertensive disorders were also reduced in the detemir group.^15^ Feig et al (*n* = 502) investigated harms and benefits of the addition of metformin (500 mg BD) compared to placebo, in women treated with a standard regimen of insulin.^25^ There was no difference in the primary outcome which consisted of fetal/neonatal adverse outcomes (metformin 40% vs. placebo 40%, *p* = 0.86, RR 1.02 [95% CI 0.83–1.26]). Secondary outcomes demonstrated that women treated with metformin achieved lower glycaemia, required less insulin, gained less weight and had a lower rate of Caesarean section compared to placebo.^25^ Infants of women treated with metformin had reduced adiposity measures and fewer larger infants, although there were more SGA infants.^25^

Furthermore, a secondary analysis of the MiTy trial^25^ investigated whether treatment with metformin had a differential effect in pregnant individuals with Type 2 diabetes plus or minus polycystic ovarian syndrome (PCOS) (*n* = 482).^26^ In a small subgroup analysis, women with PCOS and treated with metformin had a significantly higher incidence of worsening pre‐existing hypertension during pregnancy compared to those with PCOS in the placebo group and those without PCOS [PCOS metformin = 7 (16.7%) vs. PCOS placebo = 2 (4.5%) vs. no PCOS metformin = 13 (6.7%) vs. no PCOS placebo = 20 (10.1%), interaction effect 6.42, 95% CI 1.2–51.7, *p* = 0.046].^26^ Additionally, women with PCOS and treated with metformin demonstrated a significantly higher incidence of extreme LGA infants (>97th percentile) compared to those with PCOS in the placebo group and those without PCOS [PCOS metformin = 12 (28.6%) vs. PCOS placebo = 6 (14.0%) vs. no PCOS metformi*n* = 25 (13.1%) vs. no PCOS placebo = 41 (21.4%), interaction effect 6.5, 95% CI 1.72–28.5, *p* = 0.008].^26^

Two studies compared the effect of metformin to insulin on maternal and neonatal outcomes^18^, ^19^; Ainuddin et al (*n* = 250) and Refuerzo et al (pilot study; *n* = 25) aimed to compare metformin to insulin alone but in both studies, if the requirement for metformin exceeded 2500 mg, insulin was commenced.^18^, ^19^ In the RCT by Ainuddin et al, ~85% of those randomised to metformin required supplementary insulin treatment. There was a reduction in the primary outcomes of NICU admission (metformin 43.8% vs. metformin + insulin 23.3% vs. insulin 69.9%, *p* < 0.01) and neonatal hypoglycaemia (metformin 25.0% vs. metformin + insulin 7.8% vs. insulin 30.0%, *p* < 0.01) in the metformin (and metformin/insulin) treated group, as well as a reduction in a number of secondary outcomes including gestational hypertension and weight gain, and an increase in SGA infants.^18^ Refuerzo et al reported no differences between groups in their small pilot study.^19^

Two studies investigated the impact of CGM use in women with Type 2 diabetes (embedded with other types of diabetes) on fetal growth compared to self‐monitoring of blood glucose (SMBG); Secher et al intermittently utilised a first‐generation Guardian Real‐Time sensor (Medtronic, Northridge, California), recruiting individuals with both Type 1 and Type 2 diabetes between 2009 and 2011 at ≤14 weeks gestation (*n* = 154; 31 with Type 2 diabetes).^17^ Voormolen et al utilised the iPro2 (Medtronic, Northridge, California) CGM system intermittently in individuals with pre‐existing diabetes and gestational diabetes, recruiting at <16 weeks' gestation (*n* = 300; 81 with Type 2 diabetes).^20^ Neither study noted significant differences in either primary or secondary outcomes in women randomised to CGM compared to standard treatment.

A later study, Li et al, investigated the impact of intermittently scanned CGM (Freestyle Libre, Abbott) use for 2 weeks in women with Type 2 diabetes, compared to SMBG. They recruited between 2016 and 2018 at 12–14 weeks' gestation (*n* = 124). Two weeks' use resulted in improvements in time in range (TIR) (SMBG 62 ± 11% vs. CGM 69 ± 10%, *p* < 0.001) and reductions in time above range (TAR) (SMBG 31 ± 8% vs. CGM 25 ± 7%, *p* < 0.001) as well as a number of other short‐term outcomes.^24^

E‐Mekawy et al randomised women with Type 2 diabetes (*n* = 40) to moderate intensity aerobic training for 10 weeks alongside standard clinical care, to investigate the effect on umbilical artery blood flow, maternal glucose and neonatal wellbeing (Apgar score). All participants had a BMI of >30 kg/m^2^ and received insulin therapy. Of the limited outcomes assessed in this small study, differences were noted between groups after 10 weeks of moderate aerobic exercise in ultrasonographical measures of placental blood flow and maternal fasting blood glucose levels.^21^

A pilot RCT examined the feasibility of a Group Diabetes Prenatal Care programme in women with Type 2 diabetes (*n* = 38) and gestational diabetes (*n* = 78) between 22‐ and 34‐weeks' gestation compared to individual care delivery.^23^ Group delivery included twice‐weekly 2‐hourly sessions beginning with a 30‐min self‐assessment of blood pressure, weight and identification of elevated blood glucose levels, followed by 60–90 min of interactive activities exploring diabetes, pregnancy and health. Improved outcomes were identified in this pilot RCT; however, the only measure specified for women with Type 2 diabetes alone was HbA1c levels prior to delivery (post intervention) which was equivalent between groups.^23^

Min et al randomised women with Type 2 diabetes (*n* = 88) to receive 600 mg of DHA‐enriched fish oil supplementation or placebo, embedded in a larger study including women without diabetes (*n* = 173).^16^ This was based on the premise that long‐chain polyunsaturated omega‐3 fatty acid is believed to be important for fetal neuro‐visual development and is lower in women with Type 2 diabetes. DHA levels in red cell phosphatidylcholine, red cell phosphatidylethanolamine and plasma phosphatidylcholine were higher in the intervention group than the control group in the third trimester. Neonates of women in the intervention group had increased DHA in red cell phosphatidylethanolamine and plasma phosphatidylcholine; however, there was no effect on body composition of the fetus or neonate^16^ (Data S3).

#### 3.2.2 Postpartum interventions

A single RCT undertaken during the postpartum period was identified. Women with Type 2 diabetes (*n* = 69) were randomised to undertake home‐based tai chi qigong exercises five times a week for 12 weeks alongside standard diabetes care, or to standard diabetes care alone.^22^ Those completing the intervention had lower mean fasting plasma glucose levels (intervention 120.19 ± 17.51 mg/dL vs. control 129.88 ± 15.23 mg/dL, *p* = 0.02), HbA1c levels (intervention 6.83 ± 0.97% vs. control 7.70 ± 0.84%, *p* = 0.038) and systolic and diastolic blood pressure (intervention 70.40 ± 17.54 mmHg vs. control 76.50 ± 19.10 mmHg, *p* = 0.032) than those in the control group (Data S3).

### 3.3 Risk of bias and quality metrics of included studies

The risk of bias of the studies included in this systematic review is shown in Data S4. In total, only two trials were assessed as ‘low risk of bias’,^23^, ^25^ with the remaining nine trials classified as ‘high risk of bias’.^15^, ^16^, ^17^, ^18^, ^19^, ^20^, ^21^, ^22^, ^24^ The main source of bias across the studies was commonly the lack of reporting on compliance and adherence to a given intervention. Nine trials reported conducting a power calculation (Data S3),^15^, ^16^, ^17^, ^18^, ^19^, ^20^, ^22^, ^23^, ^25^ with four of these trials being underpowered either at randomisation^23^ or at analysis after losing participants at follow‐up.^15^, ^19^, ^22^ Studies were also limited by sample size, with only one large‐scale RCT identified in this review which included 502 participants.^25^

## 4 DISCUSSION

This review aimed to evaluate and synthesise RCT evidence for the optimal approach to the management of women with Type 2 diabetes in the pre‐pregnancy, pregnancy and the postpartum periods. Our findings highlight a paucity of specifically tailored interventions for women of reproductive age with Type 2 diabetes, with a particular lack of studies conducted in the pre‐pregnancy and postpartum periods. Studies primarily targeted glycaemia management, with a lack of emphasis on other aspects of optimised care. This review was also limited by studies that were inadequately designed, statistically powered or reported.

There was limited good quality RCT evidence, with most studies being small, underpowered or without a power calculation, as well as poor reporting of methodology and outcomes. However, in a well‐designed large study undertaken in women with Type 2 diabetes, Feig et al demonstrated safety, and maternal and neonatal benefits when metformin was added to a standard insulin regimen. There was no evidence of adverse fetal/neonatal events (primary outcome), with improvement in glycaemia, less insulin needed, less weight gain and fewer Caesarean sections compared to placebo, as well as reduced neonatal adiposity and LGA. There were however more SGA infants (a secondary outcome).^25^ A small subgroup analysis of this study exploring the effect of metformin in individuals with both Type 2 diabetes and PCOS suggested worse outcomes in this group; however further investigation is needed.^26^ Two studies aimed to compare metformin versus insulin for the management of hyperglycaemia but were limited either by sample size^19^ or were complicated by the need for additional insulin treatment to be added to the metformin group.^18^, ^19^ Despite this, Ainuddin et al reported maternal and neonatal benefits of metformin use, as well as an increase in SGA.^18^ A recent meta‐analysis of 21 RCTs assessing the efficacy of metformin alone or as an add‐on treatment to insulin in women predominantly with gestational diabetes (19 studies) as well as Type 2 diabetes (2 studies) similarly showed an improvement in maternal and infant outcomes and was associated with SGA infants.^27^ Current evidence supports the use of metformin during pregnancy for women with Type 2 diabetes. There are still outstanding knowledge gaps, however, with regard to longer term maternal and infant effects, as well as little innovation in this area to optimise care; assessment of new preparations or other hypoglycaemic agents is lacking.

In a medium sized comparative effectiveness trial that recruited both Type 2 diabetes and overt diabetes at <21 weeks' gestation, insulin detemir improved a composite neonatal outcome and was associated with improved maternal outcomes including a reduction in maternal hypoglycaemia and hypertensive disorders compared to NPH.^15^ Given the variety of insulin types and regimens that are available, it is surprising that this review identified only one study that evaluated differing approaches among women of reproductive age. Further studies are needed to evaluate effectiveness.

Through the international, multicentre CONCEPTT study,^28^ CGM has been shown to improve maternal and neonatal outcomes in pregnant individuals with Type 1 diabetes and has subsequently been embedded into clinical guidelines.^4^ There is scope within clinical practice to use CGM for individuals without Type 1 diabetes, which would include Type 2 diabetes, if they use insulin and experience either severe hypoglycaemia or ‘unstable blood glucose levels that are causing concern’^4^; however, current evidence for this recommendation or further use in Type 2 diabetes is lacking. This paucity of evidence was underlined by this review; included studies either used out‐of‐date technology, included only few women with Type 2 diabetes or used CGM for only a short period of time.^17^, ^20^, ^24^ In the coming years, this area will be addressed by studies such as the PROTECT trial (https://www.isrctn.com/ISRCTN12804317) and the CGM2 trial (https://reporter.nih.gov/search/7AGH82‐JnE2RTnZ4cj_sJQ/project‐details/10776545).

Dietary changes and exercise underpin management of glycaemia in pregnant women with Type 2 diabetes.^4^ Despite behavioural approaches being central to good glycaemic management, only two studies were identified, both of which were small.^21^, ^23^ The first, which introduced a moderate intensity aerobic exercise intervention concentrating on placental blood flow as an outcome, lacked methodological detail and did not report behavioural change or weight related outcomes.^21^ The second, a pilot group prenatal care programme, included little data relevant to women with Type 2 diabetes as it was embedded in a larger study.^23^ An additional study which investigated the effects of DHA‐enriched fish oil supplementation,^16^ being more mechanistic in nature, requires further research for clinical translation. High‐quality, large‐scale novel dietary and exercise interventions to optimise care and outcomes are warranted for this group.

The current systematic search revealed only one intervention study in the postpartum period; in a study of 69 randomised women undertaken in Thailand, tai chi qigong reduced fasting plasma glucose and HbA1c, as well as lowering systolic and diastolic blood pressure.^22^ However, larger studies are needed prior to implementation including replication across diverse populations. Many studies have explored postpartum interventions for the prevention of Type 2 diabetes in those with gestational diabetes^29^; however, there is an urgent need for evidence‐based effective behavioural interventions to improve interpregnancy and long‐term health in individuals with Type 2 diabetes.

No RCTs were identified in the pre‐pregnancy period, which is critical for optimal fetal development. However, a recent systematic review identified four observational studies that investigated preconception interventions, albeit with a limited impact in the uptake of care among women with Type 2 diabetes; pre‐pregnancy care was more likely to improve pregnancy preparation indicators such as achieving pregnancy glucose targets and high dose folic acid intake.^30^ An ongoing study evaluating a multi‐modal intervention targeting Primary Care infrastructure (e.g. care pathways, resources and training) to improve pregnancy preparation in women with Type 2 diabetes (the PREPARED study) will report in 2025.^31^

Finally, most studies in this review were conducted in high‐income countries,^15^, ^16^, ^17^, ^19^, ^20^, ^21^, ^23^, ^25^ with only three studies conducted in low‐income settings.^18^, ^22^, ^24^ Given the potential additional barriers for women with Type 2 diabetes in low‐income countries in accessing and receiving care, it is imperative that future RCTs in this group are conducted in low‐income settings, reaching a group who may particularly benefit from the effectiveness of novel interventions.

## 5 STRENGTHS AND LIMITATIONS

This systematic review has several strengths. To the best of our knowledge, this is the first systematic review to evaluate the optimal approach for women with Type 2 diabetes in the pre‐pregnancy, pregnancy and postpartum periods. Comprehensive search strategies were used to identify RCTs that targeted women with Type 2 diabetes, including mixed diabetes populations but with segregated Type 2 diabetes data. This review was undertaken by an international group of scientists with expertise in diabetes and women's health research following a robust methodological approach and prospective registration of the protocol on PROSPERO.

Limitations are largely as a result of the methodology, quality and characteristics of the included studies. The majority of studies included a small number of women with Type 2 diabetes, many not focusing solely on this group, impacting power and interpretation of study results. We pragmatically included studies that encompassed individuals diagnosed with ‘overt diabetes’ but acknowledge that this is a disparate group who may not have diabetes in the immediate postpartum.^15^, ^18^ Furthermore, the significant heterogeneity of interventions explored did not permit meta‐analysis and robust identification of effective management approaches. Publication bias may have been introduced through inclusion of studies only published in the English language.

## 6 CONCLUSION

As the burden of Type 2 diabetes increases in women who enter pregnancy, alongside recognised suboptimal maternal and offspring outcomes, there is an urgent need for evidence‐based interventions that improve the pregnancy planning and preparation journey, and beyond. This systematic review revealed a lack of well‐designed adequately powered studies. Co‐developed tailored pre‐pregnancy, pregnancy and postpartum interventions, including novel approaches, are urgently needed to improve outcomes for women of reproductive age with Type 2 diabetes, and their offspring. To achieve this, funding dedicated to improving care for this group should be a priority.

## FUNDING INFORMATION

D.S. is supported by the National Institute for Health and Care Research (NIHR) through an NIHR Advanced Fellowship (NIHR302955) and the NIHR Southampton Biomedical Research Centre (NIHR203319). C.L.M. is funded by Diabetes UK through a Harry Keen Intermediate Clinical Fellowship (17/0005712) and the European Foundation for the Study of Diabetes—Novo Nordisk Foundation Future Leaders' Award (NNF19SA058974). S.L.W. is supported by a grant from the Medical Research Council (UK) (MR/W003740/1).

## CONFLICT OF INTEREST STATEMENT

The authors declare no conflicts of interest.

## Supporting information


Data S1.

Data S2.

Data S3.

Data S4.
