# Supplementary material for: Interventions in women with type 2 diabetes mellitus in the pre‐pregnancy, pregnancy and postpartum periods to optimise care and health outcomes: A systematic review
Source: Diabet Med. 2024 Nov 11;42(1):e15474. doi: 10.1111/dme.15474 (PMC11635590; doi:10.1111/dme.15474)
Supplement: Supplementary file 1 — Data S1. [file DME-42-e15474-s001.docx]

**Supporting Information 1. Electronic database search strategies**

**MEDLINE**

#1. Exp Preconception Care/

#2. pre-pregnancy OR prepregnancy OR pre-conception OR preconception OR pregestation* OR pre-gestation* OR before pregnancy OR before gestation OR pregnancy plan* OR planning pregnancy OR prior to conception OR prior to pregnancy OR pregnancy intention*

#3. #1 OR #2 = 23830 results

#4. Exp Pregnancy/

#5. Exp Perinatal Care/

#6. Exp Prenatal Care/

#7. #4 OR #5 OR #6

#8. Pregnan* OR gestation* OR before delivery OR perinatal OR prenatal OR pre-natal OR antenatal OR ante-natal

#9. #7 OR #8=1214544 results

#10. Interconception OR Inter-conception OR between pregnan* OR period between pregnanc* OR interpregnancy OR inter-pregnancy OR intergestation OR inter-gestation = 4182 results

#11. exp Postpartum Period/

#12. exp Postnatal Care/

#13. #11 OR #12

#14. postpartum OR post-partum OR postnatal OR post-natal OR after pregnan* OR after gestation OR after delivery OR post pregnan* OR post-pregnan* OR post gestation OR post-gestation

#15. #13 OR #14 = 253906 results

#16. #3 OR #9 OR #10 OR #15 = 1315918 results

#17. Exp diabetes mellitus, type 2/

#18. type 2 diabetes mellitus OR Diabetes Mellitus Type 2 OR diabetes mellitus adult onset OR diabetes mellitus non-insulin dependent OR diabetes mellitus noninsulin dependent OR diabetes mellitus maturity onset OR maturity-onset diabetes mellitus OR diabetes mellitus type II OR diabetes type II OR diabetes type 2 OR type II diabetes mellitus OR type II diabet* OR T2 diabetes mellitus OR T2 diabet* OR T2D* OR type 2 diabet* OR niddm OR non-insulin dependent diabetes mellitus

#19. #17 OR #18 = 219028 results

#20. #16 AND #19 = 7496 results

#21. Limit #20 to human = 6185 results

**CINAHL**

#1. MH “Prepregnancy care”

#2. "pre-pregnancy" OR prepregnancy OR "pre-conception" OR preconception OR pregestation* OR "pre-gestation*" OR "before pregnancy" OR "before gestation" OR "pregnancy plan*" OR "planning pregnancy" OR "prior to conception" OR "prior to pregnancy" OR "pregnancy intention*"

#3. #1 OR #2 = 15455 results

#4. (MH “Pregnancy+”) OR (MH “Pregnancy in Diabetes+”)

#5. MH “Perinatal care”

#6. MH “Prenatal care”

#7. #4 OR #5 or #6

#8. Pregnan* OR gestation* OR “before delivery” OR perinatal OR prenatal OR “pre-natal” OR antenatal OR “ante-natal”

#9. #7 OR #8=319814 results

#10. Interconception OR “Inter-conception” OR “between pregnan*” OR “period between pregnanc*” OR interpregnancy OR “inter-pregnancy” OR intergestation OR “inter-gestation” = 1675 results

#11. (MH Postnatal Period+) OR (MH Postnatal Care+)

#12. postpartum OR "post-partum" OR postnatal OR "post-natal" OR "after pregnan*" OR "after gestation" OR "after delivery" OR "post pregnan*" OR "post-pregnan*" OR "post gestation" OR "post-gestation"

#13. #11 OR #12 = 63,831 results

#14. #3 OR #9 OR #10 OR #13 = 342883 results

#15. MH “Diabetes mellitus, Type 2”

#16. “type 2 diabetes mellitus” OR “Diabetes Mellitus Type 2” OR “diabetes mellitus adult onset” OR “diabetes mellitus non-insulin dependent” OR “diabetes mellitus noninsulin dependent” OR “diabetes mellitus maturity onset” OR “maturity-onset diabetes mellitus” OR “diabetes mellitus type II” OR “diabetes type II” OR “diabetes type 2” OR “type II diabetes mellitus” OR “type II diabet*” OR “T2 diabetes mellitus” OR “T2 diabet*” OR T2D* OR “type 2 diabet*” OR niddm OR “non-insulin dependent diabetes mellitus”

#17. #15 OR #16 = 86187 results

#18. #14 AND #17 = 3289 results

#19. Limit #18 to human = 1468 results

**PsycINFO**

#1. pre-pregnancy OR prepregnancy OR pre-conception OR preconception OR pregestation* OR pre-gestation* OR before pregnancy OR before gestation OR pregnancy plan* OR planning pregnancy OR prior to conception OR prior to pregnancy OR pregnancy intention* = 3433 results

#2. Exp Pregnancy/

#3. Exp Perinatal Period/

#4. Exp Prenatal Care/

#5. #2 OR #3 OR #4

#6. Pregnan* OR gestation* OR before delivery OR perinatal OR pre-natal OR prenatal OR antenatal OR ante-natal

#7. #5 OR #6= 99145 results

#8. Interconception OR Inter-conception OR between pregnan* OR period between pregnanc* OR interpregnancy OR inter-pregnancy OR intergestation OR inter-gestation = 580 results

#9. exp Postnatal Period/

#10. postpartum OR post-partum OR postnatal OR post-natal OR after pregnan* OR after gestation OR after delivery OR post pregnan* OR post-pregnan* OR post gestation OR post-gestation

#11. #9 OR #10 = 38164 results

#12. #1 OR #7 OR #8 OR #11 = 117160 results

#13. Exp Type 2 diabetes/

#14. type 2 diabetes mellitus OR Diabetes Mellitus Type 2 OR diabetes mellitus adult onset OR diabetes mellitus non-insulin dependent OR diabetes mellitus noninsulin dependent OR diabetes mellitus maturity onset OR maturity-onset diabetes mellitus OR diabetes mellitus type II OR diabetes type II OR diabetes type 2 OR type II diabetes mellitus OR type II diabet* OR T2 diabetes mellitus OR T2 diabet* OR T2D* OR type 2 diabet* OR niddm OR non-insulin dependent diabetes mellitus

#15. #13 OR #14 = 11328 results

#16. #12 AND #15 = 335 results

#17. Limit #19 to human = 295 results

**ASSIA**

#1. "pre-pregnancy" OR prepregnancy OR "pre-conception" OR preconception OR pregestation* OR "pre-gestation*" OR "before pregnancy" OR "before gestation" OR "pregnancy plan*" OR "planning pregnancy" OR "prior to conception" OR "prior to pregnancy" OR "pregnancy intention*" = 4627 results

#2. (MAINSUBJECT.EXACT.EXPLODE (“Perinatal period”) OR MAINSUBJECT.EXACT.EXPLODE(“Perinatal Care”) OR MAINSUBJECT.EXACT.EXPLODE(“Pregnancy”) OR MAINSUBJECT.EXACT.EXPLODE (“Perinatal”) OR MAINSUBJECT.EXACT.EXPLODE(“Antenatal”) OR MAINSUBJECT.EXACT.EXPLODE (“Antenatal Care”)) OR (Pregnan* OR gestation* OR “before delivery” OR perinatal OR prenatal OR “pre-natal” OR antenatal OR “ante-natal”) = 57932 results

#3. Interconception OR “Inter-conception” OR “between pregnan*” OR “period between pregnanc*” OR interpregnancy OR “inter-pregnancy” OR intergestation OR “inter-gestation” = 621 results

#4. (MAINSUBJECT.EXACT.EXPLODE(“Postnatal care”) OR MAINSUBJECT.EXACT.EXPLODE(“Postpartum women”)) OR (postpartum OR "post-partum" OR postnatal OR "post-natal" OR "after pregnan*" OR "after gestation" OR "after delivery" OR "post pregnan*" OR "post-pregnan*" OR "post gestation" OR "post-gestation") = 15316 RESULTS

#5. #1 OR #2 OR #3 OR #4= 64244 RESULTS

#6. (MAIN SUBJECT.EXACT.EXPLODE(“Type 2 diabetes mellitus”)) OR (“type 2 diabetes mellitus” OR “Diabetes Mellitus Type 2” OR “diabetes mellitus adult onset” OR “diabetes mellitus non-insulin dependent” OR “diabetes mellitus noninsulin dependent” OR “diabetes mellitus maturity onset” OR “maturity-onset diabetes mellitus” OR “diabetes mellitus type II” OR “diabetes type II” OR “diabetes type 2” OR “type II diabetes mellitus” OR “type II diabet*” OR “T2 diabetes mellitus” OR “T2 diabet*” OR T2D* OR “type 2 diabet*” OR niddm OR “non-insulin dependent diabetes mellitus”) = 8140 RESULTS

#7. #5 AND #6 = 1473 results

**Cochrane Library**

#1 MeSH descriptor: [Preconception Care] explode all trees

#2 pre-pregnancy OR prepregnancy OR pre-conception OR preconception OR pregestation* OR pre-gestation* OR “before pregnancy” OR “before gestation” OR pregnancy NEXT plan* OR “planning pregnancy” OR “prior to conception” OR “prior to pregnancy” OR pregnancy NEXT intention*

#3 #1 OR #2 = 2211 results

#4 MeSH descriptor: [Pregnancy] explode all trees

#5 MeSH descriptor: [Perinatal Care] explode all trees

#6 MeSH descriptor: [Prenatal Care] explode all trees

#7 #4 OR #5 or #6

#8 Pregnan* OR gestation* OR “before delivery” OR perinatal OR prenatal OR pre-natal OR antenatal OR ante-natal

#9 #7 OR #8 = 88266 results

#10 Interconception OR inter-conception OR between NEXT pregnan* OR period NEXT between NEXT pregnanc* OR interpregnancy OR inter-pregnancy OR intergestation OR inter-gestation = 222 results

#11 MeSH descriptor: [Postpartum Period] explode all trees

#12 MeSH descriptor: [Postnatal Care] explode all trees

#13 #11 OR #12

#14 postpartum OR post-partum OR postnatal OR post-natal OR after NEXT pregnan* OR “after gestation” OR “after delivery” OR post NEXT pregnan* OR post-pregnan* OR “post gestation” OR post-gestation

#15 #13 OR #14 = 19054 results

#16 #3 OR #9 OR #10 OR #15 = 93511 results

#17 MeSH descriptor: [Diabetes Mellitus, Type 2] explode all trees

#18 "Type 2 diabetes mellitus" OR "diabetes mellitus Type 2" OR "diabetes mellitus adult onset" OR "diabetes mellitus non-insulin dependent" OR "diabetes mellitus noninsulin dependent" OR "diabetes mellitus maturity onset" OR "maturity-onset diabetes mellitus" OR "diabetes mellitus type II" OR "diabetes type II" OR "diabetes type 2" OR "type II diabetes mellitus" OR type NEXT II NEXT diabet* OR "T2 diabetes mellitus" OR T2 NEXT diabet* OR T2D* OR type NEXT 2 NEXT diabet* OR NIDDM OR "non-insulin dependent diabetes mellitus"

#19 #17 OR #18 = 47691 results

#20 #16 AND #19 = 1703 results (1721 -3 special collections, -15 clinical answers = 1703)

**EMBASE**

#1. Exp Prepregnancy Care/

#2. pre-pregnancy OR prepregnancy OR pre-conception OR preconception OR pregestation* OR pre-gestation* OR before pregnancy OR before gestation OR pregnancy plan* OR planning pregnancy OR prior to conception OR prior to pregnancy OR pregnancy intention*

#3. #1 OR #2 = 35968 results

#4. Exp Pregnancy/

#5. Exp Perinatal Care/

#6. Exp Prenatal Care/

#7 Exp Perinatal Period/

#8 Exp Prenatal Period/

#9 Exp Pregnancy diabetes mellitus/

#10. #4 OR #5 OR #6 OR #7 OR #8 OR #9

#11. Pregnan* OR gestation* OR before delivery OR perinatal OR prenatal OR pre-natal OR antenatal OR ante-natal

#12. #10 OR #11 = 1494490 results

#13. Interconception OR Inter-conception OR between pregnan* OR period between pregnanc* OR interpregnancy OR inter-pregnancy OR intergestation OR inter-gestation = 5943 results

#14. exp Postnatal Care/

#15. postpartum OR post-partum OR postnatal OR post-natal OR after pregnan* OR after gestation OR after delivery OR post pregnan* OR post-pregnan* OR post gestation OR post-gestation

#16. #14 OR #15 = 373576 results

#17. #3 OR #12 OR #13 OR #16 = 1660828 results

#18. Exp non insulin dependent diabetes mellitus/

#19. type 2 diabetes mellitus OR Diabetes Mellitus Type 2 OR diabetes mellitus adult onset OR diabetes mellitus non-insulin dependent OR diabetes mellitus noninsulin dependent OR diabetes mellitus maturity onset OR maturity-onset diabetes mellitus OR diabetes mellitus type II OR diabetes type II OR diabetes type 2 OR type II diabetes mellitus OR type II diabet* OR T2 diabetes mellitus OR T2 diabet* OR T2D* OR type 2 diabet* OR niddm OR non-insulin dependent diabetes mellitus

#20. #18 OR #19 = 355406 results

#21. #17 AND #20 = 14642 results

#22. Limit #21 to human = 13179 results

#23. Limit #22 to (article or article in press) = 5894
