# Supplementary material for: Interventions in women with type 2 diabetes mellitus in the pre‐pregnancy, pregnancy and postpartum periods to optimise care and health outcomes: A systematic review
Source: Diabet Med. 2024 Nov 11;42(1):e15474. doi: 10.1111/dme.15474 (PMC11635590; doi:10.1111/dme.15474)
Supplement: Supplementary file 2 — Data S2. [file DME-42-e15474-s002.docx]

**Supporting Information 2. Characteristics of included randomised controlled trials**

| **Reference Publication Date**  **Study Period**  **Country of Study** | **Study Design** | **Intervention Period** | **Population** | **Aim** | **Gestational age, weeks** | **Sample Size** | **Age, years** | **Ethnicity** | **BMI, kg/m^2^ or BMI category** |
| --- | --- | --- | --- | --- | --- | --- | --- | --- | --- |
| Ainuddin et al.  2015  January 2009-January 2014  Pakistan | An active controlled, parallel-group, randomised, open label trial | Pregnancy | Women with pre-pregnancy Type 2 diabetes and newly diagnosed overt diabetes in pregnancy | To evaluate the effects of metformin therapy in  Type 2 diabetes in pregnancy and compare it with standard treatment insulin. | Metformin: 10.75 ± 5.98  Metformin and Insulin: 10.09 ± 4.86  Insulin: 9.57 ± 5.20 | Randomised  Metformin: n = 125  Insulin: n = 125  Analysed  Metformin: n = 16  Metformin and insulin: n = 90  Insulin: n = 100 | Baseline:  Metformin: 31.75 ± 2.82  Metformin and insulin: 34.09 ± 3.51  Insulin: 33.73 ± 2.95 | Not reported | Early pregnancy:  Metformin: 28.25 ± 1.98  Metformin and insulin: 33.59 ± 3.97  Insulin: 32.96 ± 4.04 |
| E-Mekawy et al.  2012 | RCT | Pregnancy | Women with Type 2 diabetes | To determine the effects of moderate aerobic antenatal exercise on the umbilical blood flow and neonate wellbeing in Type 2 diabetic pregnant women. | Eligibility criteria:  24 weeks’ gestation | N = 40 | All participants aged between 25 - 32 | Not reported | All participants with BMI > 30 |
| Feig et al.  2020  May 2011-October 2018  Australia, Canada | Randomised, parallel, double-masked, placebo-controlled trial. | Pregnancy | Women with Type 2 diabetes | To investigate whether the addition of metformin to a standard regimen of insulin, would increase or decrease the risk of neonatal morbidity and mortality in pregnant women with Type 2 diabetes. | At randomisation:  Metformin: 16.5 ± 4.0  Placebo: 16.4 ± 3.8 | Randomisation: N = 502  (Metformin: n = 253, Placebo: n = 249) | Metformin: 34.7 ± 5.0  Placebo: 35.0 ± 4.6 | Metformin:  European: 77 (30)  Non-European: 176 (70)  Aboriginal: 18 (7)  African or Caribbean origin: 40 (16)  East Asian origin: 26 (10)  Hispanic origin: 5 (2)  Middle Eastern origin: 14 (6)  Pacific Islands origin: 3 (1)  South Asian: 32 (13)  Multi-ethnic: 14 (6)  Other: 18 (7)  Unknown: 6 (2)  Placebo:  European: 75 (30)  Non-European: 174 (70)  Aboriginal: 17 (7)  African or Caribbean origin: 36 (15)  East Asian origin: 30 (12)  Hispanic origin: 7 (3)  Middle Eastern origin: 10 (4)  Pacific Islands origin: 3 (1)  South Asian: 48 (19)  Multi-ethnic: 10 (4)  Other: 11 (4)  Unknown: 2 (<1) | Metformin: 35.0 ± 7.1  Placebo: 35.2 ± 7.2 |
| Fishel et al.  2021  September 2018-January 2020  USA | RCT | Pregnancy | Women with Type 2 or overt Type 2 diabetes  mellitus (gestational diabetes diagnosed at <20 weeks’ gestation) | To determine whether treatment with insulin detemir of  pregnant women with Type 2 or overt Type 2 diabetes mellitus reduces adverse neonatal and  maternal outcomes compared with treatment with neutral protamine Hagedorn  (NPH). | Eligibility criteria:  <21 weeks' gestation | Randomisation: N = 108 (Detemir: n = 57, NPH: n = 51)  Analysed: N = 103  (Detemir: n = 53, NPH: n = 50) | Detemir: 32.1 ± 6.2  NPH: 32.5 ± 6.0 | Detemir  Non-Hispanic White: 3 (5.2)  Non-Hispanic Black: 14 (24.6)  Hispanic: 36 (63.2)  Asian: 4 (7.0)  NPH  Non-Hispanic White: 6 (11.7)  Non-Hispanic Black: 14 (27.5)  Hispanic: 31 (60.8)  Asian: 0 (0) | Detemir: 36.2 (32.5 - 40.8)  NPH: 34.2 (29.9 - 37.2) |
| Min et al.  2014  January 2008-December 2011  UK | Double-blind, placebo-controlled RCT | Pregnancy | Women with Type 2 diabetes and healthy women. | To test if docosahexaenoic acid-enriched fish oil supplementation rectifies red cell membrane lipid anomaly in pregnant women with Type 2 diabetes and their neonates and alters fetal body composition. | Fish oil: 9.9 (5.1 - 15.9)  Placebo: 10.4 (4.3 - 15.7) | Randomisation:  N=88 (Fish oil: n = 41, Placebo: n = 47) | Fish oil: 34.0 (20.0-45.0)  Placebo: 37.0 (27.0-45.0) | Fish oil:  Asian: 18 (43.9)  African/Afro-Caribbean: 15 (36.6)  Caucasian: 5 (12.2)  Other: 3 (7.3)  Placebo:  Asian: 27 (57.5)  African/Afro-Caribbean: 10 (21.3)  Caucasian: 5 (10.6)  Other: 5 (10.6) | Fish oil: 30.6 ± 5.9  Placebo: 30.4 ± 6.4 |
| Refuerzo et al.  2015  September 2009-August 2011  USA | Open-label pilot RCT | Pregnancy | Women with Type 2 diabetes | To compare the effects of metformin versus insulin on achieving glycaemic control and improving maternal and neonatal outcomes in pregnant women with Type 2 diabetes | Metformin: 16 (range 8 - 19)  No metformin: 16 (range 6 - 18) | Randomised:  N = 25 (metformin: n = 11, insulin: n = 14)  Analysed: N = 21 (metformin: n = 8, insulin: n = 13) | Metformin: 30.9 ± 5.5  Insulin: 32.3 ± 4.3 | Metformin:  African American: 3 (37.5)  Caucasian: 4 (50)  Hispanic: 0 (0)  Other: 1 (12.5)  Insulin:  African American: 4 (30.8)  Caucasian: 6 (46.2)  Hispanic: 1 (7.7)  Other: 2 (15.4) | Metformin: 35.9 ± 5.2  Insulin: 40.1 ± 8.4 |
| Secher et al.  2013  February 2009-February 2011  Denmark | RCT | Pregnancy | Women with Type 1 diabetes and Type 2 diabetes | To assess whether intermittent real-time continuous glucose monitoring improves glycaemic control and pregnancy outcome in women with pregestational diabetes. | Eligibility criteria:  <14 weeks' gestation | Randomisation:  N = 154 (n = 31 with Type 2 diabetes) | Not reported | Not reported | Not reported |
| Voormolen et al.  2018  July 2011-September 2015  Netherlands, Belgium | Open label, pragmatic, RCT | Pregnancy | Pre-existing diabetes or gestational diabetes | To evaluate the effectiveness of intermittent use of continuous glucose monitoring  in pregnant women with pregestational or gestational diabetes. | Eligibility criteria:  <16 weeks’ gestation | Randomised:  N = 300 (continuous glucose monitoring: n = 147, standard treatment: n = 153)  Type 2 diabetes at baseline:  continuous glucose monitoring: n = 40  standard treatment: n = 41 | Not reported | Not reported | Not reported |
| Carter et al.  2022  USA | RCT | Pregnancy | Women with Type 2 diabetes or gestational diabetes | To determine the feasibility of incorporating a diabetes management intervention into group prenatal care (Diabetes Group Prenatal Care) and to determine whether Diabetes Group Prenatal Care is as effective as individual prenatal care in improving engagement in diabetes self-care activities | Group care = 27.1 ± 3.5 weeks  Individual care = 26.1 ± 3.5 weeks | Group care N = 40 (n = 20 with Type 2 diabetes)  Individual care N = 38 (n = 18 with Type 2 diabetes) | Group care = 30.2 ± 4.6  Individual care = 31.0 ± 5.4 | Group care:  African-American = 16 (40.0)  White, non-Hispanic = 3 (7.5)  Hispanic = 21 (52.5)  Individual care:  African-American = 15 (39.4)  White, non-Hispanic = 3 (7.9)  Hispanic = 20 (52.6) | Group care:  Normal = 1 (2.5)  Overweight = 6 (15.0)  Obese = 33 (82.5)  Individual care:  Normal = 3 (7.9)  Overweight = 11 (28.9)  Obese = 24 (63.2) |
| Youngwanichsetha et al.  2013  Thailand | Parallel arm RCT | Postpartum | Postpartum women with a history of Type 2 diabetes | To assess the effect of tai chi qigong on  plasma glucose levels and health status of postpartum Thai women with a history of Type 2 diabetes. | Not applicable | Randomised  N = 69 (I: n = 34, C: n = 35)  Analysis  I: n = 32, C: n = 32 | I = 35.0 ± 5.63  C: 36.16 ± 4.48 | Not applicable | I = 26.77 ± 3.25  C = 27.45 ± 3.62 |
| Atkins et al.  2023  May 2011 – October 2018  Canada, Australia | Secondary analysis of RCT | Pregnancy | Women with Type 2 diabetes | 1. To determine whether the additional presence of PCOS, in the setting of Type 2 diabetes in pregnancy, would be associated with an increase in adverse maternal and fetal outcomes.  2. To examine whether the metformin effect might differ in women with PCOS from the broader population of women with Type 2 diabetes in pregnancy | Eligibility criteria: 6 and 22 weeks’ plus 6 days’ gestation | No PCOS metformin n = 198  No PCOS placebo n = 198  PCOS metformin n = 42  PCOS placebo n = 44 | No PCOS = 35.1 [31.4, 38.6]  PCOS = 35.7 [32.4, 39.0] | No PCOS: European = 113 (28.5) South Asian = 63 (15.9) African or Caribbean = 66 (16.7) East Asian = 42 (10.6) Aboriginal = 31 (7.8) Other = 20 (5.1) Middle Eastern = 18 (4.5) Multi-Ethnic = 19 (4.8) Hispanic = 10 (2.5) Pacific Islands = 5 (1.3) Jewish = 2 (0.5) Unknown = 7 (1.8)  PCOS: European = 30 (34.9) LiSouth Asian = 14 (16.3) African or Caribbean = 6 (7.0) East Asian = 14 (16.3) Aboriginal = 2 (2.3) Other = 7 (8.1) Middle Eastern = 4 (4.7) Hispanic = 2 (2.3) Pacific Islands = 0 (0.0) Jewish = 2 (2.3) Unknown = 1 (1.2) | No PCOS = 33.2 ± 7.3 PCOS = 36.0 ± 7.2 |
| Li et al.  2021  December 2016 - 2018  China | RCT | Pregnancy | Women with Type 2 diabetes | To investigate the effects of intermittently scanned continuous glucose monitoring on blood  glucose control, clinical value of blood glucose monitoring and production of urinary ketone bodies in pregestational  diabetes mellitus | Gestational age at first visit (weeks):  Control group = 9.4 ± 2.9  Intermittently scanned continuous glucose monitoring group = 9.5 ± 2.5 | Control group = 60  Intermittently scanned continuous glucose monitoring group = 64 | Control group = 30.6 ± 3.1 years  Intermittently scanned continuous glucose monitoring group = 31.2 ± 4.1 years | Not applicable | Overweight (%) at first visit ≤ BMI < 28:  Control group = 35 (58.3)  Intermittently scanned continuous glucose monitoring group = 37 (57.8)  Obesity (%) at first visit BMI ≥ 28:  Control group + 14 (23.3)  Intermittently scanned continuous glucose monitoring group = 16 (25.0) |
|  |  |  |  |  |  |  |  |  |  |

RCT = randomised controlled trial; I = intervention group; C = control group; NPH = neutral protamine Hagedorn

Note: Data presented as *n* (%) for categorical variables, mean ± standard deviation, median [interquartile range (IQR)] or median [10^th^ – 90^th^ centile] for continuous variables.
