# Supplementary material for: Interventions in women with type 2 diabetes mellitus in the pre‐pregnancy, pregnancy and postpartum periods to optimise care and health outcomes: A systematic review
Source: Diabet Med. 2024 Nov 11;42(1):e15474. doi: 10.1111/dme.15474 (PMC11635590; doi:10.1111/dme.15474)
Supplement: Supplementary file 3 — Data S3. [file DME-42-e15474-s004.docx]

**Supporting Information 3. Outcomes and results for included randomised controlled trials**

| **Reference Publication Date**  **Study Period**  **Country of Study** | **Intervention** | **Outcomes** | **Results** | **Power calculation** |
| --- | --- | --- | --- | --- |
| Ainuddin et al.  2015  January 2009-January 2014  Pakistan | Women were randomised to either metformin or insulin.  Metformin was started at dose of 500 mg/day orally and increased up to 2500 mg in three divided doses as tolerated by the participant and until glycaemic control was achieved. Target blood glucose levels for glycaemic control were taken as fasting blood glucose (FBS) ≤ 100 mg/dL (5.5 mmol/lit) and postprandial blood glucose levels (1.5 hours after meals) of ≤126 mg/dL (7 mmol/lit). If target blood glucose levels were not maintained anytime during treatment even after maximum dose of metformin; insulin was added as supplementary treatment with metformin.  Insulin (Humulin R, Humulin N (Lilly)) was prescribed as a combination of short acting and intermediate acting human insulin as twice daily injections before meals in morning (before breakfast) and in evening (before dinner) to cover for the three meals and three snacks a day or as multiple injections of short acting insulin before meals and intermediate acting insulin at bedtime depending on the individual requirement, in order to achieve the desired glycaemic targets. Dose of insulin was calculated according to body weight and  gestational age.  Participants were given dietary advice according to BMI and provided with iron, calcium, vitamin B12, and folic acid supplements. | Primary outcomes: alive baby, neonatal hypoglycaemia requiring intravenous dextrose therapy, and NICU admissions >24 hours.  Secondary outcomes: birth weight, LGA/macrosomia, SGA, neonatal morbidity like transient tachypnoea of newborn, respiratory distress syndrome, prematurity, sepsis, jaundice, and birth trauma, maternal glycaemic control, weight gain in pregnancy, maternal hypertensive complications including pregnancy induced hypertension and preeclampsia, and treatment compliance, dose, and cost of metformin and insulin treatment. | Primary outcomes:  NICU admissions: Metformin alone = 43.8% vs. metformin and insulin = 23.3% vs. insulin = 69%, *p* ≤ 0.01.  Neonatal hypoglycaemia: Metformin alone = 25% vs. metformin and insulin = 7.8% vs. insulin = 30%, *p* ≤ 0.01  Secondary outcomes:  Weight gain: Metformin alone = 10.38 kg ± 1.2 vs. metformin and insulin = 10.52 ± 1.14 kg vs. insulin = 11.80 ± 0.86 kg, *p* ≤ 0.01.  Pregnancy-induced hypertension: Metformin alone = 6.2% vs. metformin and insulin = 23.3% vs. insulin = 36%, *p* = 0.020.  Caesarean section: Metformin alone = 81.2% vs. metformin and insulin = 52.2% vs. insulin = 82% in insulin group, *p* ≤ 0.01.  SGA: Metformin alone = 31.2% vs. metformin and insulin = 14.4% vs. insulin = 2%, *p* ≤ 0.01. | Using Chi-square for proportion with 95% CI, sample size of 206, and effect size of 0.233 with 2 degrees of freedom, the power is calculated at 0.86 for the primary outcome measure, neonatal hypoglycaemia, and for NICU admission, the power of the study is calculated at 0.99 using an effect size of 0.296. |
| E-Mekawy et al.  2012 | Group A: moderate intensity aerobic training (60% of maximum heart rate) 30 minutes 3 times/week for 10 weeks (30 sessions) using an electronic bicycle ergometer in addition to their medical treatment. Every session of the exercise training programme (30 minutes) consisted of three stages: First stage (warming up), consisted of 5 minutes warming up in the form of pedalling at a speed of 60 revolutions per minutes without load. Second stage (Active stage) consisted of 20 minutes pedalling at the same speed of the first stage with adjusted load to achieve 60% of maximal heart rate (maximal heart rate= 220-age of the woman). Third stage (Cooling down), which is the same as first stage.  Group B: follow medical treatment.  All participants were on insulin therapy. | Umbilical artery blood flow: maximum systolic and end diastolic velocities of the umbilical artery for which S/D ratio, RI and PI were calculated, and 1- and 5-min Apgar score.  Primary and secondary outcomes not specified. | Umbilical artery maximum systolic velocity: Group A = 0.397 ± 0.016 m/h vs. Group B = 0.423 ± 0.018, *p* = 0.004.  Umbilical artery S/D ratio: Group A = 2.535 ± 0.066 vs. Group B = 3.052 ± 0.057, *p* = 0.001  Umbilical artery RI: Group A = 0.608 ± 0.009 vs. Group B = 0.674 ± 0.007, *p* = 0.001  Increase in 1-min Apgar in infants from group A compared to group B, *p* < 0.05  Blood glucose level: Group A = 94.550 ± 13.372 vs. Group B =140.250± 12.192, *p* < 0.001 | No power calculation reported. |
| Feig et al.  2020  May 2011-October 2018  Australia, Canada | Women were randomly assigned to metformin or placebo in addition to a standard regimen of insulin, which usually included short-acting insulin before meals and long-acting insulin before bed. | Primary outcome: composite of fetal and neonatal outcomes including one or more of the following: pregnancy loss (miscarriage, termination, stillbirth, or neonatal death up to 28 days), preterm birth, birth injury, moderate or severe respiratory distress syndrome, neonatal hypoglycaemia, and NICU admission lasting >24 h.  Secondary maternal outcomes: glycaemic control (HbA1c and capillary glucose), hypertensive disorders, caesarean section, gestational weight gain, and insulin dose.  Secondary neonatal outcomes: individual outcomes in the composite, birthweight measures (LGA, SGA, birthweight (≥4000 g), cord blood C-peptide, neonatal adiposity outcomes (fat mass, length, and flank skin fold; and skinfold thicknesses), gestational age at birth, length of infant hospital stay. | Secondary maternal outcomes:  Metformin-treated women achieved better glycaemic control (HbA1c at 34 weeks’ gestation 41.0 mmol/mol [SD 8.5] vs C = 43.2 mmol/mol [–10]; 5.90% vs 6.10%; *p* = 0.015; mean glucose 6.05 [0.93] mmol/L vs 6.27 [0.90] mmol/L; difference –0.2 [–0.4 to 0.0])  Metformin-treated women required less insulin (1.1 units per kg per day vs C = 1.5 units per kg per day; difference –0.4 [95% CI –0.5 to –0.2]; *p* < 0.0001),  Metformin-treated women gained less weight (7.2 kg vs C = 9.0 kg; difference –1.8 [–2.7 to –0.9]; *p* < 0.0001)  Metformin-treated women had fewer caesarean births (125 [53%] of 234 vs C =148 [63%] of 236; relative risk [RR] 0.85 [95% CI 0.73 to 0.99]; *p* = 0.031)  Secondary neonatal outcomes:  Metformin-exposed infants weighed less (mean birthweight 3156 g [SD 742] vs 3375 g [742]; difference –218 [–353 to –82]; *p* = 0.002)  Fewer were above the 97th centile for birthweight (20 [9%] in the metformin group vs 34 [15%] in the placebo group; RR 0.58 [0.34 to 0.97]; *p* = 0.041) fewer weighed 4000 g or more at birth (28 [12%] in the metformin group vs 44 [19%] in the placebo group; RR 0.65 [0.43 to 0.99]; *p* = 0.046), and  Metformin-exposed infants had reduced adiposity measures (mean sum of skinfolds 16.0 mm [SD 5.0] vs 17.4 [6.2] mm; difference –1.41 [–2.6 to –0.2]; *p* = 0.024; mean neonatal fat mass 13.2 [SD 6.2] vs 14.6 [5.0]; *p* = 0.017)  30 (13%) infants in the metformin group and 15 (7%) in the placebo group were SGA (RR 1.96 [1.10 to 3.64]; *p* = 0.026) | To have 80% power to detect a 25% relative risk reduction (12.5% absolute reduction), at a two-sided significance level of 5%, the authors calculated that 492 participants would be needed, and account for loss to follow-up, aimed for 500 participants. |
| Fishel et al.  2021  September 2018-January 2020  USA | Participants were randomly allocated in 1:1 ratio to receive either insulin detemir or NPH prescriptions. In the insulin detemir group, the total daily dose of insulin was divided into 50% insulin detemir and 50% short-acting insulin. Based on clinician’s preference, insulin detemir dose could be divided in half and then injected in the morning and evening, 12 hours apart. Short-acting insulin was divided with one-third each being injected with breakfast, lunch, and dinner meals.  In the NPH insulin group, approximately 60% of the total daily dose was given in the morning and 40% in the evening. For the total morning dose, two-thirds were NPH, and one-third was the short-acting insulin. The total evening dose was halved: the short acting insulin injected at dinner, whereas the NPH was injected at bedtime.  At least 1 meeting with a registered dietitian and diabetes care and education specialist to learn how to self-administer insulin and were followed up in a Maternal-Fetal Medicine clinic. | Primary outcome: composite of adverse neonatal complications and consisted of 1 or more of the following: shoulder dystocia, LGA, NICU admission, neonatal hypoglycaemia, respiratory distress.  Neonatal secondary outcomes: Gestational age at delivery, SGA, 5-minute Apgar score of <7, lowest glucose level, need for intravenous glucose, respiratory distress syndrome, need for mechanical ventilation or CPAP, neonatal jaundice requiring therapy, brachial plexus injury, and hospital length of stay.  Secondary maternal outcomes: hypoglycaemic events, antepartum hospital admission for glucose control, hypertensive disorder of pregnancy, maternal weight gain, caesarean delivery, and postpartum complications, such as wound infection and endometritis. | Primary outcome:  87% posterior probability of reduced primary outcome with detemir compared with NPH (Bayesian posterior adjusted relative risk reduction 0.88; 95% Credible interval, 0.61-1.12).  Secondary outcomes:  Reduced maternal hypoglycaemic events with detemir (97% probability of benefit; Bayesian posterior adjusted relative risk reduction 0.59; 95% Credible interval 0.29-1.08) and hypertensive disorders (88% probability of benefit; Bayesian aRR 0.81; 95% Credible interval 0.54-1.16). | A sample size of 108 provided 80% power to identify the most effective treatment, assuming the NPH group had a true adverse outcome rate of 43% and the true adverse rate was 29% in the detemir group (33% reduction). |
| Min et al.  2014  January 2008-December 2011  UK | Women were randomly assigned to receive either DHA-enriched fish oil or placebo (high oleic acid sunflower oil) and instructed to take two capsules per day until delivery. Two capsules of fish oil provided 600 mg of DHA. The fish oil capsule contained 43.7% of DHA and 7.5% eicosapentaenoic acid, whereas oleic acid comprised 82.6% of the placebo. Both supplements contained vitamin E as an antioxidant and encapsulated in an identical oblong soft gelatine capsule (750 mg in size). | Primary outcome: red cell DHA level of the women at the beginning of the third trimester and at delivery, and of the neonates at delivery  Secondary outcomes: fetal biometric and  neonatal anthropometric measurements (head circumference, femur, and humerus length, biparietal and occipito-frontal diameter, mid-arm and mid-thigh lean mass, mid-arm and mid-thigh fat mass, mid-thigh and abdominal circumference, and abdominal fat mass), newborn weight and length. | Analysed fish oil n = 28 vs. placebo n = 30:  Primary outcome:  Higher percentage of docosahexaenoic acid in red cell phosphatidylethanolamine in the third trimester (I = 12.0% ± 1.87 vs. C = 8.85 ± 1.82%, *p* = 0.000) and at delivery (10.7 ± 3.32% vs. 7.41 ± 2.67%, *p* = 0.001)  Neonates of women with Type 2 diabetes supplemented with fish oil had increased docosahexaenoic acid in the red cell phosphatidylethanolamine (I = 9.23 ± 1.83% vs. C = 7.74 ± 1.58%, *p* = 0.027) and plasma phosphatidylcholine (I = 6.10 ± 1.52% vs. C = 4.68 ± 1.59%, *p* = 0.020) | The sample size was calculated based on an observation from the authors’ previous study. In this study, the authors found that the DHA level in red cell phosphatidylcholine during the third trimester was 3.5% in pregnant women with Type 2 diabetes and 5.5% in those without diabetes. The authors wanted to test if supplementation with 600 mg of DHA would increase the level (3.5%) by 50% to 5.3%. The power calculation indicated that a minimum of 24 subjects per group (fish oil vs. placebo) at the third trimester will be required to detect the increase with 85% power. We also included the same number of pregnant women without diabetes in order to assess the effectiveness of supplementation. The sample size and power calculation was performed using G*Power 3 and based on a two-independent-groups, two-tailed t-test with an alpha of 0.05. |
| Refuerzo et al.  2015  September 2009-August 2011  USA | Women were randomly assigned to either metformin or  insulin.  Metformin: Metformin 500 mg daily was initiated, and women returned for routine clinical prenatal visits weekly. If greater than 50% of the glucose values were abnormal, metformin was increased to 500 mg twice a day. The metformin regimen was increased by 500 mg as needed for a maximum dose of 2,500 mg a day. Once glycaemic control was achieved, the participant followed up every 2 weeks. If the participant required greater than 2,500 mg of metformin a day without achieving glycaemic control, she was considered “failed metformin therapy” and was started on insulin, but still resumed and continued metformin. Women receiving metformin before pregnancy resumed the dose at which they were on at the start of pregnancy and increased according to the above protocol.  Insulin: regimens were based on maternal weight and gestational age. The total insulin dose was divided into: AM dose into two-thirds NPH and one-third regular insulin, PM dose into half NPH and half regular insulin. Insulin doses were increased or decreased 10 to 20% according to SBGM. Women receiving insulin before pregnancy resumed or switched to an equivalent insulin regimen.  All participants received prenatal care through the high-risk diabetic clinic. The ADA diet was recommended based on weight and instructions on self-blood glucose monitoring four times a day (fasting and 2 hours postprandial) were provided. Instruction for exercise was also provided. | Primary outcome: rate of women with an HbA1c < 7% at the time of delivery.  Secondary outcomes: change in HbA1c, maternal, and neonatal complications including preeclampsia, failed metformin therapy, caesarean delivery rate, macrosomia, shoulder dystocia, respiratory distress syndrome, or need for neonatal dextrose therapy. | No significant differences between groups. | The authors reviewed recent deliveries at their institutions of women with Type 2 diabetes and noted a 60% rate of women achieving a HbA1c less than 7% at the time of delivery. If there were 50 women in each group with an effect size of 50%, a *p*-value < 0.05, the power would be 0.87. |
| Secher et al.  2013  February 2009-February 2011  Denmark | Women were randomised to intermittent real-time CGM for 6 days at the first pregnancy visit at 8 weeks and at 12, 21, 27 and 33 weeks, on top of routine pregnancy care. The women were encouraged to use real-time CGM continuously, especially in cases of hypoglycaemia unawareness.  The women were instructed to continue performing self-monitored plasma glucose measurements as recommended and to verify the accuracy of real-time CGM glucose values with self-monitored plasma glucose measurement before making management decisions. At real time CGM alarms with subsequent plasma glucose <4.0 mmol/L, the women were advised to supplement carbohydrate intake. Real-time CGM alarms for hyperglycaemia were tackled on an individual basis, including physical exercise like walking or supplementary rapid-acting insulin. All women received a scheduled phone call the day after initial sensor insertion.  At first pregnancy visit, all women had a dietitian appointment for individual dietary planning following national guidelines for diabetes diet.  After each monitoring period, downloaded real-time CGM data were printed out, with hard copies given to both the participants and health professionals. Each real-time CGM reading was discussed with a diabetes caregiver using locally developed guidelines. The primary focus was glycaemic trends during night-time with emphasis on the prevention of hypoglycaemia. Thereafter, hypoglycaemia and pre and postprandial glucose values during the daytime were evaluated, aiming for glucose values between 4.0 and 8.0 mmol/L. Therapeutic adjustments in diet, exercise, and insulin doses were primarily based on self-monitored plasma glucose values, in combination with real-time CGM data. | Primary outcome: LGA infants  Secondary outcomes: preterm delivery and/or severe neonatal hypoglycaemia.  Other pregnancy outcomes: miscarriage (before 22 weeks), preeclampsia, birthweight SD score, neonatal hypoglycaemia, and major congenital malformation | No significant differences in glycaemic control, insulin doses, maternal and perinatal outcomes in women with Type 2 diabetes | Based on the assumption that the prevalence of LGA infants was 50% in the study population and that the use of real-time CGM could reduce it to 20%, and a type 1 error of 5% and a type 2 error of 20%, the number of patients needed in each arm was 45. |
| Voormolen et al.  2018  July 2011-September 2015  Netherlands, Belgium | Women allocated to CGM were instructed to use the device for 5-7 days every 6 weeks. Glucose profiles were obtained retrospectively, directly after each use, and were evaluated by the local endocrinologist. Insights were discussed with the individual and changes in diet or insulin therapy were advised.  All participating women performed SMBG (4-8 times/day; at least fasting, after every meal, at bedtime and, preferably, also before every meal). They were provided with specific targets: 3.5-5.3 mmol/L for fasting state; ≤ 7.8 mmol/L 1 hour after meals; ≤ 6.7 mmol/L 2 hours after meals. HbA1c levels were measured every 4 weeks throughout pregnancy. Additional obstetric and diabetes care was provided according to local protocols and national guidelines. | Primary outcome: macrosomia.  Secondary maternal endpoints: pregnancy induced hypertension, pre-eclampsia, HELLP syndrome, elevated liver enzymes and low platelet count, caesarean section, severe hypoglycaemia, HbA1c levels.  Secondary neonatal endpoints: birthweight, extremely large size for gestational age, SGA, preterm birth, neonatal mortality, birth trauma, neonatal hypoglycaemia, culture proven sepsis, respiratory distress syndrome, bronchopulmonal dysplasia, intraventricular haemorrhage, necrotising enterocolitis, major congenital malformations. | No difference in macrosomia, gestational hypertension, pre-eclampsia, HELLP syndrome between the group | The study was powered to detect a reduction in macrosomia from 45% to 30%, anticipated to outweigh the cost of CGM use. Taking into account a possible 10% protocol violation and drop-out rate, we needed a total of 300 women (150 in both arms) (Alpha-error, .05; Beta-error, .20; one-sided test). |
| Carter et al.  2022  USA | Women randomised to Diabetes Group Prenatal Care attended the next scheduled group session after randomisation, such that women that women could enter the group at any session in the four-session “revolving door” curriculum and had the potential to complete the entire curriculum more than once, which assured adequate group sizes of at least four to six women on average. Group sessions occurred bi-weekly, lasted 2 hours, including between 2 and 10 women, and were facilitated by a Certified Nurse Midwife, Obstetrician, or Maternal Fetal Medicine physician and a co-facilitator. Women measured and recorded their weigh and circled elevated values on their blood sugar logs. The individual assessment period for each group lasted approximately 30 minutes, and could include diabetes-friendly snacks, self-reflection activities, crafts (e.g., making rice socks that could be heated for stress relief) or rotating through stations with prespecified themes relating to pregnancy or behavioural health. The remaining 60 to 90 minutes of the sessions involved the group exploring diabetes, pregnancy, and behavioural health topics through fun and interactive activities using adult learning principles. Before 37 weeks, women in Diabetes Group Prenatal Care could also be seen individually if a complication emerged that the obstetric provider felt required closer follow-up. After 37 weeks, women in Diabetes Group Prenatal Care were seen individually in the institution’s routine resident diabetes clinic during alternating weeks when the group did not meet, per the ACOG recommended prenatal visit schedule. Participants in both arms of the study were scheduled for a 6-week postpartum visit.  Women randomised to individual care received prenatal care in the diabetes clinic attended by residents and faculty physicians. Women were seen every 2 weeks, or more at the discretion of the provider, until 37 weeks of gestation and then weekly until delivery per ACOG recommendations. Visits were 10 to 20 minutes and focused on routine screening tests, review of blood sugar logs, and medication titration as needed.  Regardless of the model of prenatal care, treatment goals were to maintain a fasting blood sugar <95 mg/dL and 1-hour postprandial blood sugar <140 mg/dL. Women with type 2 diabetes were generally managed with insulin, and those with GDM received glyburide, metformin, or insulin if > 50% of blood glucose measures at any point were above the aforementioned goals.  Participants with Type 2 diabetes had their HbA1c measured at the first study visit and the last study visit before delivery (~38 weeks of gestation) | Primary outcome: Toobert's Diabetes Self-Care Activities Measure score, including self-reported assessment of nutrition, exercise, blood sugar testing, and adherence to prescribed medications based on the number of days engaging in each activity over the previous week  Secondary outcomes: hypertensive disorders of pregnancy included gestational hypertension; preeclampsia; haemolysis, elevated liver enzymes, and low platelet count syndrome; and eclampsia, gestational weight gain, rate of weight gain, breastfeeding at hospital discharge and 6 weeks postpartum (self-reported), patient satisfaction score reflecting the degree to which they felt their prenatal care met their needs on a 10-point scale (10 being most satisfied and 0 being least satisfied)  Neonatal outcomes: Gestational age, birthweight percentile, large for gestational age, small for gestational age, shoulder dystocia, neonatal hypoglycaemia, NICU admission | Secondary outcomes:  Baseline HbA1c group care = 7.7 ± 2.1 vs. individual care = 8.0 ± 1.9, *p* = 0.73  Final HbA1c before delivery group care = 6.3 ± 0.7 vs. individual care = 6.8 ± 0.9, *p* = 0.09 | The authors estimated that 84 patients would provide 90% power to detect a 1.5-day (~20%) difference in mean number of days per week engaging in diabetes self-care activities, assuming α = 0.05 and 10% loss to follow-up. |
| Youngwanichsetha et al.  2013  Thailand | Participants randomised to the control group were given standard diabetes care while those in the intervention group received standard diabetes care in addition to practising tai chi qigong exercise. The women in the intervention group were trained to perform tai chi qigong exercise over three 50-minute sessions. After the training, participants were encouraged to continue tai chi qigong exercise at home five times a week for 12 weeks. The protocol was composed of three steps: (1) warm-up exercise with 25 movements for 15 min; (2) tai chi qigong following set one of Lin Housheng’s style comprising 18 movements for 30 min; and (3) cooling down with five qigong movements for 5 min. | Primary outcomes: fasting plasma glucose, HbA1c.  Secondary outcomes: blood pressure, body weight, BMI. | Primary outcomes:  Postpartum women with Type 2 diabetes who practised tai chi qigong for 12 weeks had significantly lower fasting plasma glucose, HbA1c and blood pressure than those in the control group  Fasting plasma glucose was lower in the intervention group compared to control: 120.19 mg/dl (SD = 17.51) vs 129.88 mg/dl (SD = 15.23), *p* = 0.02  HbA1c was lower in the intervention group compared to control: 6.83% (SD = 0.97) vs 7.70% (SD = 0.84), *p* = 0.038)  Secondary outcomes:  Blood pressure was lower in the intervention group compared to control: systolic blood pressure 114.60 mmHg (SD = 16.41) vs 127.60 mmHg (SD = 18.75, *p* = 0.016 and diastolic blood pressure: 70.40 mmHg (SD = 17.54) vs 76.50 mmHg (SD = 19.10), *p* = 0.032 | The sample size per group was 2c/delta2+1, where delta was the standardised effect size; that is, the value of the mean difference of the experimental group and control group divided by the common standard deviation. A two-sided test, with the significance level set at 0.05, was employed. Power for the study was 80%. Using data from a pilot study, delta was set at 0.7. The calculated sample size per group was 33, with a total sample size of 66. |
| Atkins et al.  2023  May 2011 – October 2018  Canada, Australia | Women with Type 2 diabetes were randomised to receive either 1g metformin twice daily or placebo, in addition to their usual insulin therapy | Maternal outcomes: Gestational weight gain, final HbA1c in pregnancy at 34 - 38 weeks' gestation, total insulin dose at 34 - 36 weeks (units per kg per day), C-section, primary C-section, any hypertensive disorder, gestational hypertension, worsening chronic hypertension and pre-eclampsia.  Fetal outcomes: pregnancy loss, spontaneous abortion, stillbirth, termination, neonatal death < 28 days, live births, preterm births (< 37 weeks), birth injury, respiratory distress syndrome, neonatal hypoglycaemia, NICU admission > 24 h, gestational age at birth, large for gestational age, small for gestational age, cord blood C-peptide, shoulder dystocia, hyperbilirubinemia, congenital anomalies, length of hospital stay and composite fetal outcomes of ≥ one of the following: pregnancy loss (miscarriage, termination, stillbirth, or neonatal death), preterm birth, birth injury, respiratory distress, neonatal hypoglycaemia or NICU admission lasting > 24 h.  Primary and secondary outcomes not specified. | Worsening chronic hypertension during pregnancy: No PCOS metformin = 13 (6.7%) vs. no PCOS placebo = 20 (10.1%) vs. PCOS metformin = 7 (16.7%) vs. PCOS placebo = 2 (4.5%), interaction effect 6.42, 95% CI = 1.2 - 51.7, *p* = 0.046  Extreme LGA, ≥ 97th centile: No PCOS metformin = 25 (13.1%) vs. no PCOS placebo = 41 (21.4%) vs. PCOS metformin = 12 (28.6%) vs. PCOS placebo = 6 (14.0%), interaction effect 6.5%, 95% CI 1.72 - 28.5%) *p* = 0.008 | No power calculation reported, although authors state that study did not have sufficient power to detect small but important differences in rare adverse neonatal outcomes, such as stillbirth, termination, or birth injury. |
| Li et al.  2021  China | Participants were randomly divided into two groups. The control group used self-monitoring of blood glucose to monitor blood glucose, whilst the intermittently scanned CGM group used an intermittently scanned CGM system to monitor blood glucose. The capillary blood glucose was monitored eight times a day (before meals and 2 h after meals, before going to bed, and at midnight). Participants downloaded the Dnurse App (Beijing Dnurse Technology Co., Ltd) in which they used the Outpatient mode to upload data their postprandial glucose self-measurements measured using the Dnurse blood glucose meter. Blood glucose data were compiled into tables and charts that were then uploaded and transmitted to the Doctor’s version of Dnurse App. Intermittently scanned CGM group participants were monitored by intermittently scanned CGM (Free-Style Libre, Abbott). At the first visit, a professional diabetes nurse provided the participants with one-on-one equipment installation and system maintenance training. Glucose readings and glucose trends were also obtained by scanning the sensor with a scanning detector. Participants wore the device for a total of 14 days, during which blood sugar values were uploaded through the diabetes management APP. | The value of glycated albumin at baseline and 15th day, the value of glycated haemoglobin at baseline, hypoglycaemia, time in target during 1st and 2nd weeks, time > 7.8 mmol/L during 1st and 2nd week, time < 3.5 mmol/L during 1st and 2nd week, ketonuria positive during 1st and 2nd week, minimum blood sugar values at 0 clock (or 0:00 - 2:59), minimum blood sugar values before and after breakfast (or 3:00 - 5:59 and 6:00 - 8:59), maximum blood sugar values before and after breakfast, minimum blood sugar values before and after lunch (or 09:00 - 11:59 and 12:00 - 14:59), maximum blood sugar values before and after lunch, minimum blood sugar values before and after dinner (or 15:00 = 17:59 and 18:00 - 20:59, maximum blood sugar values before and after dinner, minimum and maximum blood sugar values before and after sleeping (or 21:00 - 23:59)  Primary and secondary outcomes not specified. | Glycaemic control characteristics and ketonuria of control group vs. intermittently scanned continuous glucose monitoring group:  The value of glycated albumin at baseline (%) = 18.6 ± 3.5 vs. 18.9 ± 3.6, *p* = 0.852  The value of glycated albumin at 14th day (%) = 16.8 ± 2.7 vs. 14.6 ± 2.2, *p* = < 0.001  The value of glycated haemoglobin at baseline (%) = 7.1 ± 0.3 vs. 7.2 ± 0.4, *p* = 0.796  Hypoglycaemia (%) = 2 (3.3%) vs. 3 (4.6%), p = 0.656  Time in target during the 1st week (3.5–7.8 mmol/L) (%) = 54 ± 10 vs. 55 ± 11, *p* = 0.703  Time in target during the 2nd week (3.5–7.8 mmol/L) (%) = 62 ± 11 vs. 69 ± 10, *p* = < 0.001  Time > 7.8 mmol/L during the 1st week (%) = 36 ± 7 vs. 35 ± 6, *p* = 0.723  Time > 7.8 mmol/L during the 2nd week (%) = 31 ± 8 vs. 25 ± 7, *p* < 0.001  Time < 3.5 mmol/L during the 1st week (%) = 8 ± 3 vs. 9 ± 4, *p* = 0.689  Time < 3.5 mmol/L during the 2nd week (%) = 6 ± 2 vs. 4 ± 3, *p* = 0.092  Ketonuria positive during the 1st week (%) = 55 ± 7 vs. 52 ± 6, *p* = 0.698  Ketonuria positive during the 2nd week (%) = 54 ± 5 vs. 42 ± 5, *p* = < 0.001  Comparison of cases with maximum and minimum blood sugar values of control group vs. intermittently scanned continuous glucose monitoring group:  Minimum values at 0 clock, n (%) = 5 (8.3) vs. minimum values during 0:00–2:59, n (%) = 20 (31.2)  Maximum values at 0 clock, n (%) = 3 (5.0) vs. maximum values during 0:00–2:59, n (%) = 0 (0)  Minimum values before breakfast, n (%) = 14 (23.3) vs. minimum values during 3:00–5:59, n (%) = 17 (26.6)  Maximum values before breakfast, n (%) = 0 (0) vs. maximum values during 3:00–5:59, n (%) = 0 (0)  Minimum values after breakfast, n (%) = 2 (3.3) vs. minimum values during 6:00–8:59, n (%) = 2 (3.12)  Maximum values after breakfast, n (%) = 14 (23.3) vs. maximum values during 6:00–8:59, n (%) = 26 (40.6)  Minimum values before lunch, n (%) = 24 (40.0) vs. minimum values during 9:00–11:59, n (%) = 8 (12.5)  Maximum values before lunch, n (%) = 0 (0) vs. maximum values during 9:00–11:59, n (%) = 12 (18.7)  Minimum values after lunch, n (%) = 2 (3.3) vs. minimum values during 12:00–14:59, n (%) 3 (4.68)  Maximum values after lunch, n (%) = 24 (40) vs. maximum values during 12:00–14:59, n (%) = 7 (11.0)  Minimum values before dinner, n (%) = 7 (11.7) vs. minimum values during 15:00–17:59, n (%) = 8 (12.5)  Maximum values before dinner, n (%) = 0 (0) vs. maximum values during 15:00–17:59, n (%) = 12 (18.7)  Minimum values after dinner, n (%) = 1 (1.7) vs. minimum values during 18:00–20:59, n (%) = 3 (4.68)  Maximum values after dinner, n (%) = 17 (28.3) vs. maximum values during 18:00–20:59, n (%) = 7 (11.0)  Minimum values before sleeping, n (%) = 5 (8.3) vs. minimum values during 21:00–23:59, n (%) = 3 (4.68)  Maximum values before sleeping, n (%) = 2 (3.3) vs. maximum values during 21:00–23:59, n (%) = 0 (0) | No power calculation reported. |

I = intervention group; C = control group; NICU = neonatal intensive care unit; LGA = large for gestational age; SGA = small for gestational age; RI = resistive index; PI = pulsatility index; S/D ratio = systolic/diastolic ratio; SD = standard deviation; NPH = neutral protamine Hagedorn; SMBG = self-monitoring of blood glucose; CGM = continuous glucose monitoring, CI = confidence interval, DHA = docosahexaenoic acid
