# Supplementary material for: Interventions in women with type 2 diabetes mellitus in the pre‐pregnancy, pregnancy and postpartum periods to optimise care and health outcomes: A systematic review
Source: Diabet Med. 2024 Nov 11;42(1):e15474. doi: 10.1111/dme.15474 (PMC11635590; doi:10.1111/dme.15474)
Supplement: Supplementary file 4 — Data S4. [file DME-42-e15474-s003.docx]

**Supporting Information 4. ‘Risk of bias’ summary**

| Quality assessment criteria | | | | | | | |
| --- | --- | --- | --- | --- | --- | --- | --- |
| Reference | A | B | C | D | E | F | Overall Bias |
| Fishel et al. 2021 |  |  |  |  |  |  |  |
| Feig et al. 2020 |  |  |  |  |  |  |  |
| Ainuddin et al. 2015 |  |  |  |  |  |  |  |
| Refuerzo et al. 2015 |  |  |  |  |  |  |  |
| Min et al. 2014 |  |  |  |  |  |  |  |
| Secher et al. 2013 |  |  |  |  |  |  |  |
| Voormolen et al. 2018 |  |  |  |  |  |  |  |
| Li et al. 2021 |  |  |  |  |  |  |  |
| E-Mekawy et al. 2012 |  |  |  |  |  |  |  |
| Carter et al. 2022 |  |  |  |  |  |  |  |
| Youngwanichsetha et al. 2013 |  |  |  |  |  |  |  |

Risk of bias legend

(A) Bias arising from the randomization process - Selection bias

(B) Bias due to deviations from intended interventions (assignment)

(C) Bias due to deviations from intended interventions (adherence)

(D) Bias due to missing outcome data - Attrition bias

(E) Bias in measurement of the outcome - Measurement bias

(F) Bias in selection of the reported result - Reporting bias

Low risk of bias

Some concerns

High risk of bias
